# The Succession of Bacterial Community Attached on Biodegradable Plastic Mulches During the Degradation in Soil

**DOI:** 10.3389/fmicb.2021.785737

**Published:** 2021-12-24

**Authors:** Zhicheng Ju, Xiongfeng Du, Kai Feng, Shuzhen Li, Songsong Gu, Decai Jin, Ye Deng

**Affiliations:** ^1^CAS Key Laboratory for Environmental Biotechnology, Research Center for Eco-Environmental Sciences, Chinese Academy of Sciences, Beijing, China; ^2^College of Resources and Environment, University of Chinese Academy of Sciences, Beijing, China

**Keywords:** biodegradable plastic mulches, plastic, bacteria, plastisphere, succession of microbiota, biodegradation, soil

## Abstract

Despite the increasing application of biodegradable plastic mulches (BDMs) in agriculture, the colonization and succession of the attached microbial community on BDMs during their degradation processes remain poorly characterized. Here, we buried four types of commonly used BDMs, including pure polylactic acid (PLA), pure polybutylene adipate terephthalate (PBAT), and two mixtures of PLA and PBAT (85:15 and 15:85 w/w), and one classic polyethylene (PE) mulch in soil for 5 months. Both plastic components and incubation time significantly shaped the β-diversities of microbiota on the plastic mulches (*p* < 0.001). Meanwhile, the microbial compositions and community structures on BDMs were significantly different from PE mulch, and when excluding PE mulch, the microbiota varied more with time than by the composition of the four BDMs. The orders *Burkholderiales* and *Pseudonocardiales* were dominant on most BDMs across different time points. The genus *Ramlibacter* was revealed as a common biomarker for both PLA and PBAT by random-forest model, and all biomarkers for the BDMs belonged to the dominant order *Burkholderiales*. In addition, degradation-related and pathogen-related functional taxa were enriched in all mulches among all 40 functional groups, while surprisingly, potential pathogens were detected at higher levels on BDMs than PE. For community assembly on all mulches, the drift and dispersal processes played more important roles than selection, and in particular, the contribution of stochastic drift increased during the degradation process of BDMs while selection decreased, while the opposite trend was observed with PE mulch. Overall, our results demonstrated some degradation species and pathogens were specifically enriched on BDMs, though stochastic processes also had important impacts on the community assembly. It suggested that, similar to conventional plastic mulch, the increased usage of BDMs could lead to potential hazards to crops and human health.

## Introduction

As a globally applied agricultural practice, plastic mulching has produced huge productive and economic advantages including increased yield and improved crop quality since 1960s (Lamont, 2005). It is estimated that the global agricultural films market used for greenhouses, mulching and silage will grow by 59% from 2018 to 2026, especially, plastic mulching account for over 40% (Sintim and Flury, 2017; Sintim et al., 2020). Unfortunately, their improper treatment and environmental accumulation have threatened the development of green and sustainable agriculture in various ways, such as biological accumulation in the food chain, metabolism of soil carbon and nitrogen, and becoming a hotspot of antibiotic resistance genes (ARGs) and pathogens (Ju et al., 2021; Zhu et al., 2021). Biodegradable plastic mulches (BDMs) were introduced in 1990s and have been developed as important substitution for classic plastic mulches in alleviating environmental pollution and reducing labor (Kasirajan and Ngouajio, 2012). Nowadays, global bioplastics production capacity is growing at a considerable rate and is expected to increase from around 2.11 million tons in 2018 to 2.62 million tons in 2023 (Chinthapalli et al., 2019). They usually include two types according to raw materials, namely, bio-based polymers represented by polylactic acid (PLA) and fossil-sourced polymers represented by polybutylene adipate terephthalate (PBAT; Kasirajan and Ngouajio, 2012). As a major advantage, BDMs are usually tilled into soil after their usage and are expected to be gradually biodegraded *in situ* over a relatively short period. Generally speaking, microorganisms acting on different types of BDMs secrete extracellular hydrolases, which break down the complex polymers into simpler oligomeric and monomeric units. Then, the microorganisms uptake and utilize them for biomass formation and energy production (Sander, 2019; Bandopadhyay et al., 2020). However, the true degradation rate and state of different types of BDMs debris in soil after harvest and their potential ecological risk in comparison with classic plastic mulches have always been controversial (Sintim and Flury, 2017; Bandopadhyay et al., 2018; Sridharan et al., 2021). Meanwhile, it has always been a key issue of concern that microorganisms preferentially adhered to the debris surface may affect the assembly processes of biofilms and lead to a series of ecological heterogeneity (Hou et al., 2021; Sun et al., 2021).

As a new anthropogenic substrate, hydrophobic plastic surfaces can readily be colonized by various microorganisms, constituting a diverse and unique ecological habitat called the “plastisphere” (Zettler et al., 2013; Amaral-Zettler et al., 2020). This region is usually considered as a hot spot for nutrient accumulation, active microbial metabolism, and biodegradation of complex compounds. Moreover, the plastisphere often contain specific functional taxa, particularly the consortia capable of degrading plastics and invasive or pathogenic species. It not only hints at the potential to isolate degradable functional taxa from them, but also raises concerns of ecological risk with respect to aquaculture and human health (Wu et al., 2019; Hou et al., 2021). Therefore, it is of great importance to study the temporal dynamics of plastisphere (Jacquin et al., 2019; Amaral-Zettler et al., 2020; Khan et al., 2020). Through whole-genome sequencing, Bhagwat et al. (2021) investigated the composition and functions of microbes attached to four kinds of plastics. The results suggested that both non-degradable plastics and a kind of BDMs exhibited unique microbial profiles and niche partitioning. All substrates were significantly enriched several microbial genera with functional genes involving in carbon cycling, xenobiotic compound degradation, and plant–pathogen interaction. Recently, a few studies carried out in soils showed similar conclusions as those from aquatic environments. The surface of PE could also act as a distinct microbial habitat in soil and select unique microbiota for enhancing pathways of amino acid metabolism and xenobiotics biodegradation (Huang et al., 2019; Ruthi et al., 2020). Zhou et al. (2021) investigated the microplastisphere of a biodegradable polymer, and the results indicated that the microbial community could utilize the polymer as a carbon source, which had a more active microbial biomass and increased specific microbial growth rate compared with that of the bulk soil. Additionally, considering that various compounds are adsorbed and released during the process of biodegradation, it was expected that a variety of biomarker taxa could be identified to distinguish microbiota between different plastic components (Zettler et al., 2013; Ruthi et al., 2020). For example, PLA/PBAT is degraded into intermediates with different chain lengths or small molecules, such as lactic acid and butanediol, which could be regarded as a nutrient source and may recruit specific microbial taxa (Zumstein et al., 2018). However, it is noteworthy that although the above studies tended to indicate that microbes selectively colonize the plastisphere and form specific biofilms, the dominance of stochastic processes in the community construction on plastics was observed in both aquatic and soil environments (Li et al., 2021a; Zhang et al., 2021; Zhu et al., 2021). Polymer types and sizes, sampling site, or exposure time may all be important factors causing higher stochasticity (Zhang et al., 2021). For example, Zhu et al. (2021) found that specific metabolic pathways selectively drove diverse ecological processes, such as the enrichment of potential pathogens and ARGs in soil, while neutral processes dominated plastisphere community assembly. Overall, current studies have focused on either traditional plastic in aquatic environments or their effects on soil microorganisms and enzyme activities (Sander, 2019; Shruti and Kutralam-Muniasamy, 2019). Little is known about the succession and assembly process of the microbial community during the degradation processes of different plastics, especially BDMs, in soils.

In order to ensure the repeatability and comparability, a pot experiment within a greenhouse was carried out in our study. PE mulch and four types of BDMs were buried into ceramic pots with 12 biological duplications and extracted for five consecutive months. We chose not only pure PLA and PBAT, but also two mixtures of these compounds by considering real agricultural application. Base on the experimental design, we proposed to address the following four scientific questions. (i) Regarding differences in composition of the microbial communities, does time or composition of the plastic have a greater impact? (ii) What kind of dynamic changes occur in the composition and functions of the attached microbiota during the degradation period, and what are their potential ecological risks? (iii) What are the biomarker taxa in the plastisphere to distinguish BDMs with PE mulch? and (iv) What are the assembly processes of microbial communities colonized on the plastic films, and how do they change over time?

## Materials and Methods

### Plastics and Soil

In addition to PE mulch, four BDMs including pure PLA, pure PBAT, and two PLA/PBAT blends (85/15 and 15/85, w/w%) were purchased from Dong Guan Universe Eco Tech Co., LTD., China. Variabilities in physicochemical properties of PLA/PBAT and their blends with different proportions have been well understood relatively. Previous studies had shown that PLA and PBAT have completely different degradation mechanisms and their blends showed an intermediate degradation behavior compared to the pure polymers in general (Jiang et al., 2006; Weng et al., 2013; Palsikowski et al., 2018). The above five types of plastics were selected in this study because they are widely used in global agricultural mulching (Franca et al., 2019). Next, all mulches were cut into square pieces of 5 cm^2^ and sterilized in an ultraviolet clean bench for 30 min to minimize microbial contamination before burial in soil (Huang et al., 2019). This method can not only simulate the state before the plastic mulches are buried in soil to some degree, but also avoid the potential dissolution of biodegradable plastic components caused by alcohol disinfection (Sanchez and Collinson, 2011). The experimental soil was agricultural surface layer soils (0–20 cm) collected from a suburban field for growing vegetables outside of Beijing (40°3′44.83″N, 116°13′23.87″E). According to the survey, no plastic mulch had ever been applied in this field, which effectively avoids the interference of plastic debris or plastic pollution on microorganisms in the original soil. The collected soil was transported to the laboratory for air drying at room temperature and sieving through a 2-mm mesh. The physicochemical properties of the raw soil were as follows: pH (8.05, 22°C), total nitrogen (TN) 547.74 mg/kg, ammonia nitrogen ( ${\mathrm{NH}}_{4}^{+}$ -N) 52.87 mg/kg, and available phosphorus (AP) 54.54 mg/kg.

### Pot Experiment and Sampling

After sieving, the soil samples were mixed evenly by hand to homogenize, and then weighed (2.5 kg) into ceramic pots. Five types of mulches were buried vertically into separate pots and all treatments were performed in 12 replicates (Supplementary Figure S1). Then, the pots were put in the condition-controlled room at 23°C ± 3°C with 15 ± 5% air moisture. The water content of the soil was adjusted every other day to maintain it constant (~15%) during the incubation period (Huang et al., 2019). To explore the development and succession of microbial communities colonized in four BDMs and PE, the mulch samples rather than soil were carefully collected from the pot for five consecutive months for a total of 300 samples. During this incubation period, the BDMs had undergone obvious morphological changes (Supplementary Figure S1).

### DNA Extraction and Amplicon Sequencing of 16S Rrna Gene

All plastic mulches collected from the soil were immediately rinsed three times with distilled water in an ultraviolet clean bench. Then, each mulch sample was cut into tiny pieces with sterile scalpels and used to extract DNA directly (Bandopadhyay et al., 2020; Bhagwat et al., 2021). Total DNA was extracted using the MoBio Power Soil DNA isolation kit (MoBio Laboratories, Carlsbad, CA, United States) according to the manufacture’s protocol. Extracted DNA was amplified using the 16S rRNA universal primer set, 515 forward (5′-GTGCCAGCMGCCGCGGTAA-3′) and 806 reverse (5′-GGACTACHVGGGTWTCTAAT-3′), that targeted the V4 hypervariable regions of the prokaryotic 16S rRNA genes and were supplemented with sample-specific barcodes at both 5′ ends (Yarza et al., 2014). The polymerase chain reaction (PCR) amplification was carried out in a 50 μl reaction system containing 0.5 μl Taq DNA Enzyme (TaKaRa), 1.5 μl dNTP mixture, 1.5 μl of both 10 μm forward and reverse primers, 2 μl of template DNA (10–30 ng), 5 μl 10× PCR buffer, and 38 μl ddH_2_O. The thermal cycling conditions were as follows: 94°C for 1 min, 35 cycles of 94°C for 20 s, 57°C for 25 s, and 72°C for 45 s, with an extension at 72°C for 10 min (Li et al., 2021b). The PCR products were purified by gel electrophoresis and quantified with Qubit fluorimeter (Invitrogen, Carlsbad, CA), and then, all the samples were sent for sequencing on the Illumina NovaSeq platform with 2*250 bp sequencing kit at Magigene Biotechnology Co., Ltd. (Guangzhou, China).

### Bioinformatic Analysis

The raw 16S rRNA gene sequences were demultiplexed using an established sequence analysis pipeline,^1^ which was integrated with various bioinformatics tools (Feng et al., 2017). The reads were assigned to different samples according to their barcodes, allowing for a single mismatch. Then, the barcode and primer sequences were trimmed. Both forward and reverse reads of the same sequence were merged using FLASH (Magoč and Salzberg, 2011). Quality control criteria consisting of average quality score > 20, minimum length of 140 bp, and no ambiguous bases were used. The zero-radius OTUs (zOTU), which are a form of amplicon sequence variants (ASVs), were generated using UNOISE 3 algorithm; then, chimera and low-abundance sequences (*n* < 8) were discarded. To eliminate the influence of differences in sequencing depth on downstream analyses, all the samples were randomly resampled to the same total number of reads (24,159). Taxonomic assignment of representative sequences was carried out with the Ribosomal Database Project (RDP) classifier based on SILVA database (Elmar et al., 2007; Wang et al., 2007). The resampled ASV table was used for subsequent community analysis.

Shannon-Wiener (H) and Richness indices were used to evaluate the alpha diversity of all samples. To visualize the underlying driving forces of microbial community variation in our data, we used principal coordinates analysis (PCoA) in combination with permutational multivariate analysis of variance (PERMANOVA) on Bray-Curtis dissimilarities among the samples. The top 40 features of Functional Annotation of Prokaryotic Taxa (FAPROTAX v.1.2.4) were used to create a functional heatmap (Louca et al., 2016). We then manually selected degradation-related and pathogen-related functional groups to create a temporal heatmap during 5 months and performed the corresponding response ratio analysis for the groups at 95% confidence intervals. The Random-Forests (RF) algorithm was carried out to identifying the most distinguish taxa of PE/PLA and PE/PBAT (Liaw and Wiener, 2002; Statnikov et al., 2008). Significantly enriched or depleted ASVs were designated as biomarkers and the RF package v.4.6–14 was used with default parameters (Zhang et al., 2019).

### Analysis of Bacterial Community Assembly Mechanism

The Infer Community Assembly Mechanisms (iCAMP) was recently reported to assess the specific process of ecological assembly and relative contributions during microbial succession trajectories (Ning et al., 2020). It provided an improved performance with higher precision and specificity compared with previous approaches, such as Quantifying assembly Processes based on Entire-community Null model analysis (QPEN). Specifically, iCAMP divided taxa into different phylogenetic bins to ensure adequate phylogenetic signal to infer selection from phylogenetic diversity. Then, the processes of selection (homogeneous selection and variable selection), dispersal (homogenizing dispersal and dispersal limitation), and drift dominating each bin were determined according to the deviation of observed taxonomic and phylogenetic diversity (Stegen et al., 2015). Finally, the relative abundance of bins governed by each process was aggregated to evaluate its influence on the entire-community assembly. The ASV table was applied to ecological null model. Our analysis was performed by using the “iCAMP version v1.3.2” with recommended default values of the Galaxy platform pipeline.^2^ We separately evaluated the relative influences of selection, dispersal, and drift in both BDMs and PE over the 5 months testing period. The turnover of three ecological processes was also shown by calculating the values of the corresponding samples in two adjacent sampling months. Scripts employed in the computational analyses are available at: https://github.com/yedeng-lab/the_microbiota_of_biodegradable_plastic.

## Results

### Different Factors Shape the Diversities of Microbiota in Various Plastics

The morphological characteristics of these mulches displayed obvious degradations in all BDMs and just some minor changes in PE mulch at the end of incubation (Supplementary Figure S1). A total of 7,029,946 high-quality reads were obtained after quality control and were grouped into 24,159 ASVs among the 300 samples. The four types of BDMs showed a larger fluctuation than PE over time in richness and Shannon indices (Supplementary Figure S2), suggesting the α-diversity of microbiota on BDMs could be more variable than those on PE mulch.

**Figure 1:**
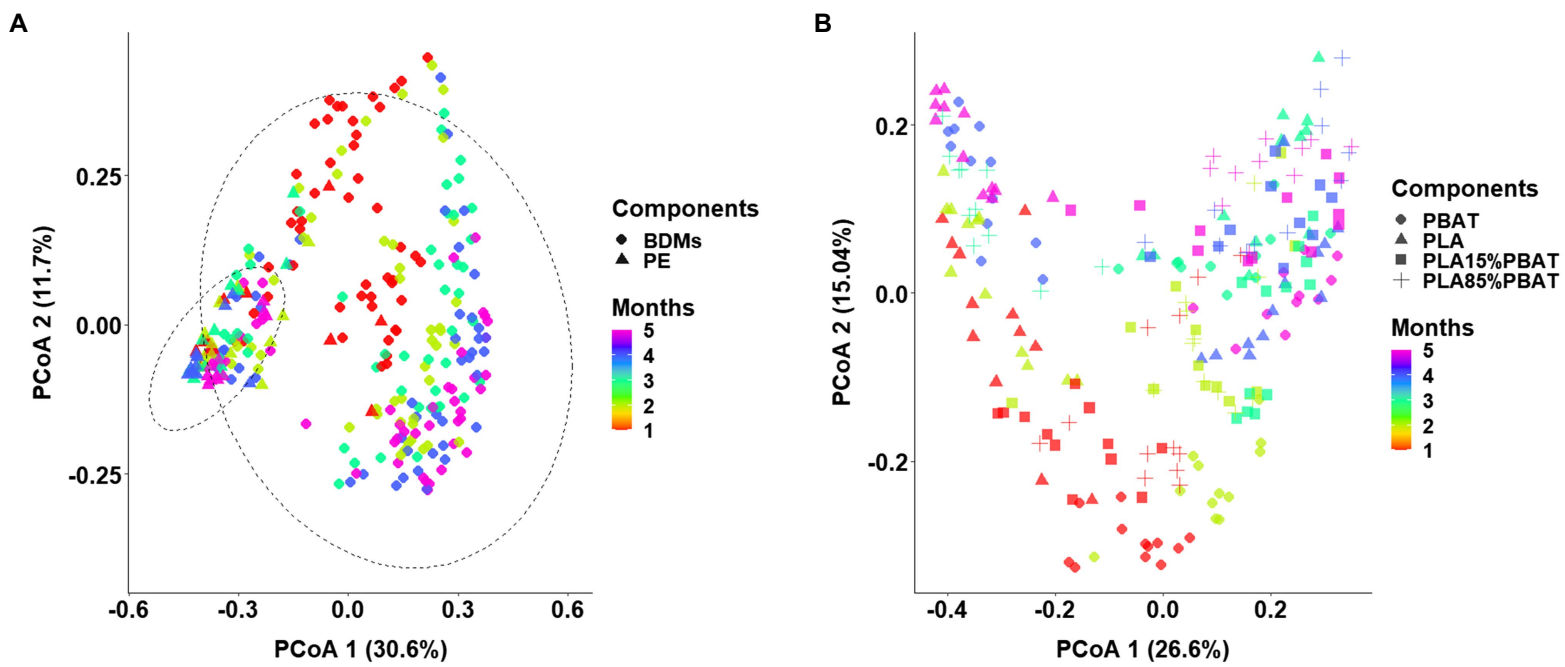
Differences in β-diversity between plastic-associated microbiota under three grouping methods, visualized by principal coordinate analysis (PCoA) based on Bray-Curtis distance. Samples separated into two groups consisting of BDMs (rounded diamonds) and PE (triangles) **(A)**. After exclusion of PE samples, PCoA analysis shows that the microbiota of four types of BDMs shifted over the 5 months in soil **(B)**. The five colors represent the 5 months of sampling **(A,B)**.

Principal coordinates analysis plot based on Bray–Curtis dissimilarity was performed to investigate the shift in β-diversity of the microbial communities (Figure 1), and PERMANOVA was used to determine the difference in variables between various groups (Table 1). Generally, clear separations were observed along the first and second PCoA axes in two groups which explained 42.3 and 41.6% of total variance, respectively (Figures 1A,B) and the dissimilarities of corresponding microbiota were all significant (Figure 1; Tables 1, *p* < 0.001). This suggested the community compositions of the five mulches were strongly affected by both sampling time and mulch component. However, it is worth noting that the impact of material was greater than that of time as the main factor between the microbiota of BDMs and PE (Table 1, *R*^2^ of components 0.1942 > *R*^2^ of months 0.0607 in group 1). Interestingly, by excluding PE mulch, among the four types of BDMs, the microbiota differentiation caused by time was greater than that caused by material composition (Table 1, *R*^2^ of months 0.0922 > *R*^2^ of components 0.0683). The above results indicated that both time and mulch composition shaped the structure of the microbial communities, but within the four types of BDMs, the microbiota could vary more significantly during the degradation process due to the composition of the mulch material.

**Table 1:** The results of PERMANOVA base on Bray-Curtis.

Groups	Factors	F. model	*R* ^2^	*p*
Group 1	Months	24.9329	0.0607	0.001
Four BDMs *vs* PE	Components	19.9219	0.1942	0.001
Group 2	Months	25.9340	0.0922	0.001
Four BDMs	Components	6.4063	0.0683	0.001

### The Composition and Succession of Bacteria Varied With Different Plastic Mulches

**Figure 2:**
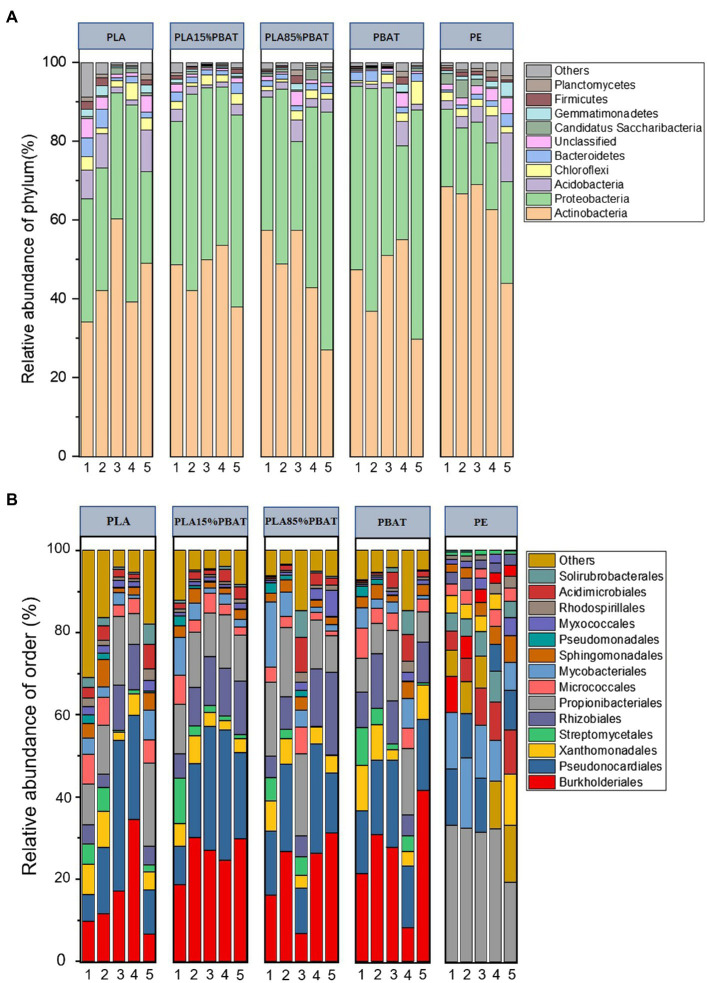
Relative abundance of bacterial phyla and order over 5 months. Stacked bar chart of the top 10 bacterial phyla **(A)** and the top 14 bacterial orders **(B)** with the largest mean relative abundance. The numbers 1–5 represent 5 months, respectively **(A,B)**.

The dominant bacteria phyla were *Actinobacteria*, *Proteobacteria*, *Acidobacteria*, *Chloroflexi*, and *Bacteroidetes* in all five plastic mulches (Figure 2A), which together encompassed 80–95% of the bacterial reads. Unlike phylum level, the four types of BDMs and PE showed totally different dominant orders (Figure 2B), where *Burkholderiales* and *Pseudonocardiales* accounted for 15–60% in BDMs and *Propionibacteriales* accounted for 20–30% in PE mulch over the 5 months period. It was clear that the microbiota of BDMs showed more dramatic dynamic changes over time at both the phylum and order levels compared to those of PE (Figure 2). In addition, as an important and widely studied plant pathogen in agricultural production (Bayer-Santos et al., 2019), *Xanthomonadales* had been found on all plastic films. In detail, it consistently ranked third at the order level for all four BDMs, while it was progressively enriched in PE during the 5 months (Figure 2B).

Although the taxonomic composition of species at the order level was similar among the four types of BDMs, there were some differences in the trend over time. *Pseudonocardiales* and *Rhizobiales* showed an increasing trend, while the proportion of *Streptomycetales* gradually decreased over time. Similarly, we further observed the higher microbial fluctuations in the PLA component (PLA/ PLA15%PBAT) than the PBAT component (PBAT/PLA85%PBAT). It was reported that degradation ease among the three components was PLA > PBAT > PE (Weng et al., 2013). Therefore, we speculated that the fluctuation of plastic-associated microbiota may be closely related to the plastic composition. In other words, the more easily degradable the plastic is, the more drastic the integral volatility of microbiota is.

### Selective Enrichment of Plastic Degradation and Pathogen-Related Functions

**Figure 3:**
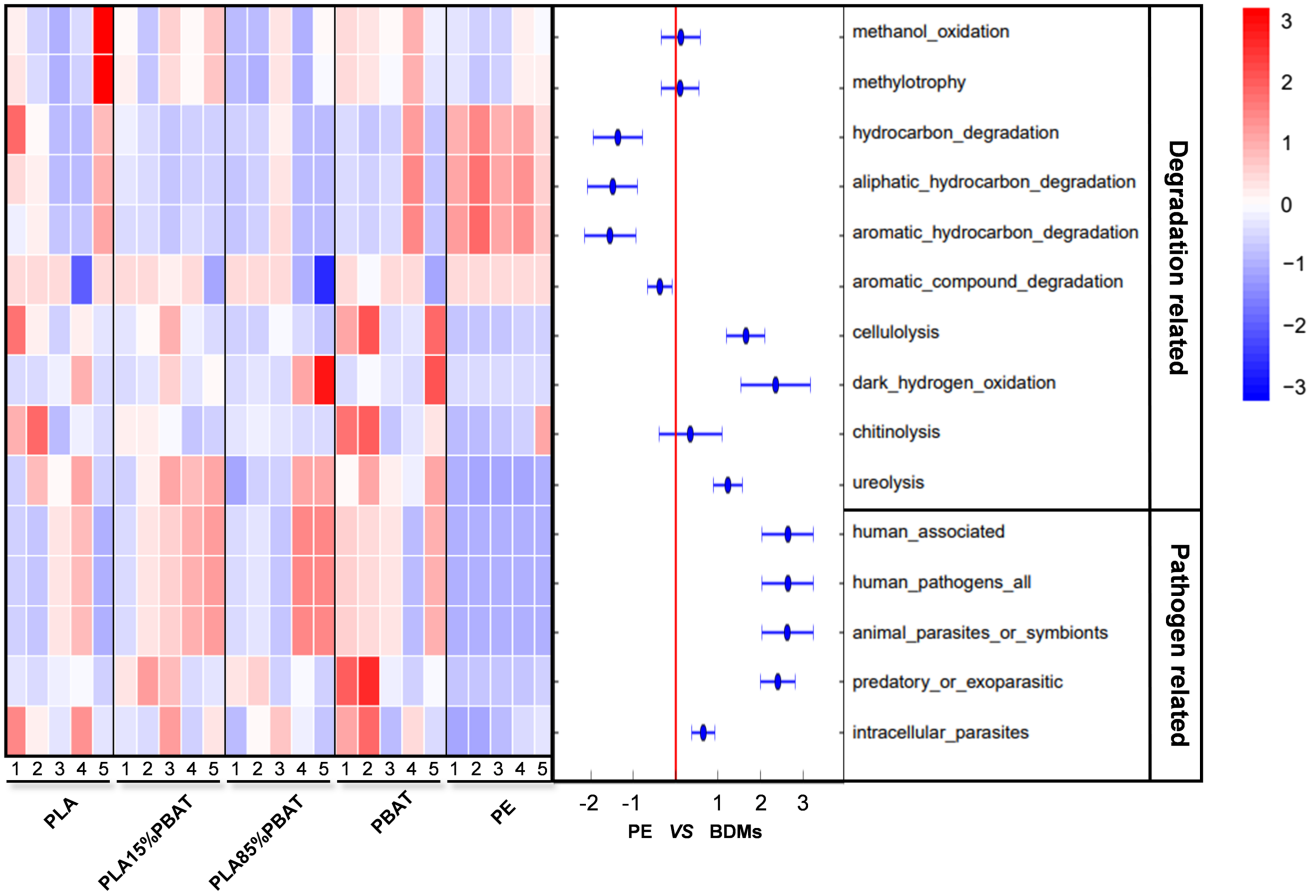
The degradation and pathogen-related functional profiles of four kinds of BDMs and PE. Degradation-related (10 types) and pathogen-related (5 types) functional categories based on FAPROTAX were used to create a temporal heatmap during 5 months and each column of the heat map represents a month. The corresponding results of response ratio at 95% confidence interval between BDMs and PE. The numbers 1–5 represent 5 months, respectively.

To further explore the functional classification of plastisphere and the dynamic succession, FAPROTAX was carried out and all functional predictions of the five plastic samples over time were presented (Supplementary Figure S3). According to the 16S rRNA gene sequence annotations, a total of 58 functional groups were obtained and 3,605 ASVs (14.92% of the total ASVs) could be assigned to at least one functional group. It was obvious that the functional distribution and temporal variation of PE and the four kinds of BDMs differed in general. The functions of carbon, nitrogen, and sulfur-related material metabolism were relatively active in the plastisphere (Supplementary Figure S3), suggesting potential impacts of plastic debris on farmland fertility and soil biogeochemical cycling. In addition, degradation-related and pathogen-related taxa were enriched in all samples to varying degrees and were extracted to make a time-scale heatmap (Figure 3). We then performed a significance analysis using response ratio at the 95% confidence interval. The results showed that although both PE and BDMs samples contained the degradation-related and pathogen-related taxa, there was a clear preference in the selection of functional groups (Figure 3). For example, cellulolysis, dark hydrogen oxidation, and ureolysis were significantly higher in BDMs than in PE, while three hydrocarbon degradation groups and an aromatic compound degradation group had higher abundance in PE during 5 months. This supported that PE and PLA/PBAT had different degradation mechanisms and recruited specific degrading bacteria.

It was noteworthy that all five functional groupings related to pathogens in all four types of BDMs had more significant enrichment than those in PE, including human associated, human pathogens all, animal parasites or symbionts, predatory or exoparasitic, and intracellular parasites. Furthermore, the first three functional taxa mentioned above, which are closely related to human pathogens, tend to increase gradually with resident time in soils, especially in the two blends, while the others showed a decreasing trend over time in BDMs. In general, most of these pathogen-associated functional groupings are types closely related to animals and humans, indicating that PLA/PBAT components may be more susceptible to colonization by potential pathogenic microorganisms in agriculture (Figure 3).

### The Distinctive Biomarkers Were Revealed by a Random-Forests Model

**Figure 4:**
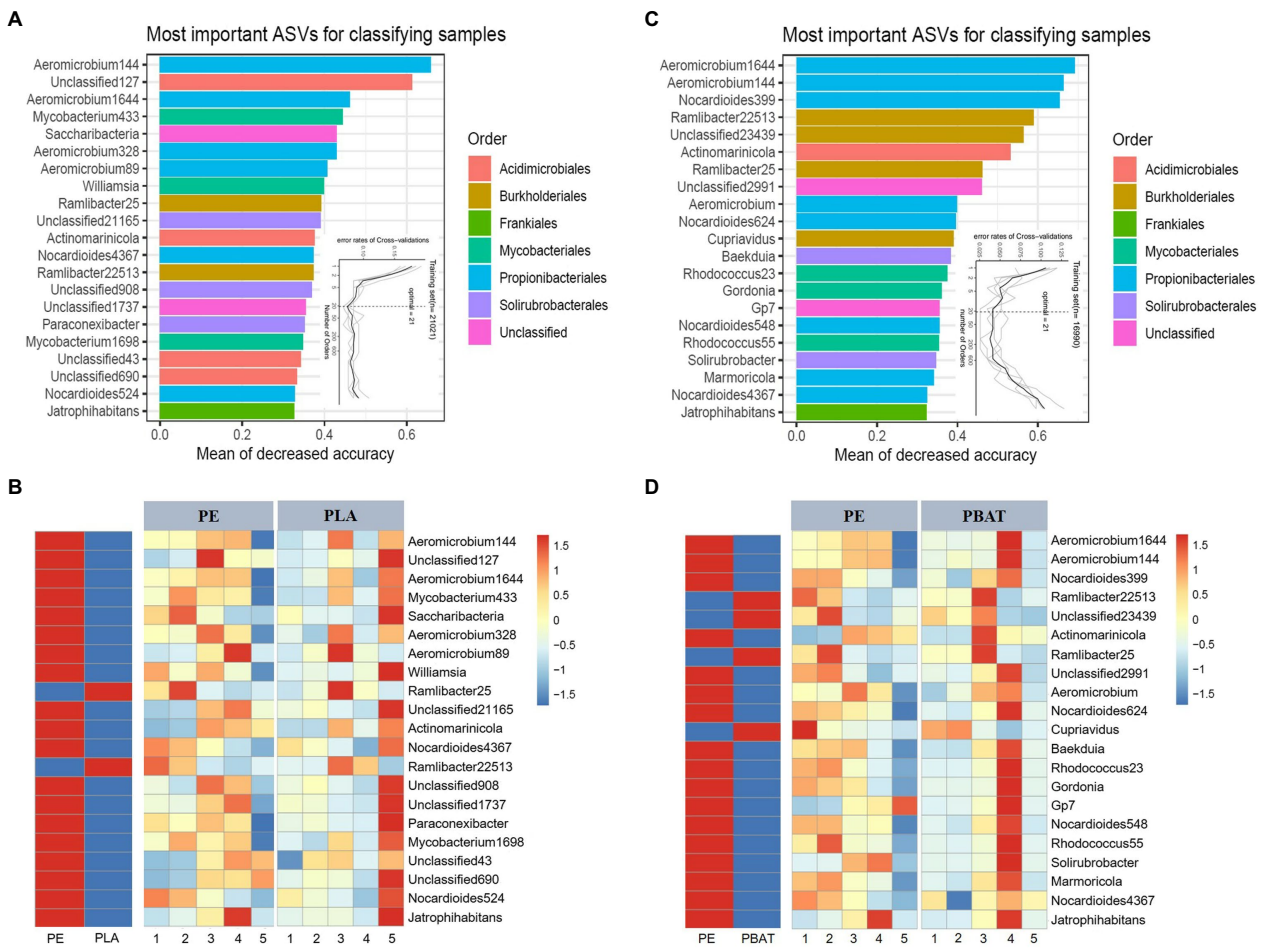
Biomarkers of plastic-related microbiota between PLA/PBAT and PE. The top 21 biomarker bacterial ASVs are accurately identified by Random-forest model in PE/PLA **(A)** and PE/PBAT **(B)**. The biomarkers are ranked in descending order of importance to the accuracy of the model. The inset represents 10-fold cross-validation error as a function of the number of input ASVs used to differentiate PE and PLA/PBAT microbiota ranked by variable importance. The corresponding heatmaps show the relative abundances of the top 21 biomarkers over 5 months for the plastics **(C,D)**.

To acquire the best discriminant performance of taxa across different plastic components, we regressed the relative abundance of bacteria using the RF machine learning algorithm to establish a model to correlate plastic microbiota composition during the 5 months incubation (Liaw and Wiener, 2002; Zhang et al., 2019). The model explained 95.90 and 92.62% of the microbiota variance related to PE/PBAT and PE/PLA separately at the order level, and the 21 most important ASVs were designated as biomarkers both in PE/PBAT and PE/PLA (Figures 4A,C). We next sought to characterize how the biomarkers changed in abundance during plastic degradation over the course of 5 months and visualized this by heatmaps. It is evident that all the most important ASVs both in PE/PBAT and PE/PLA belong to the same five orders except the unclassified. While the above orders show a wide range in relative abundance (Figures 2B, 4), indicating that PE and BDMs differ in both abundant and rare species. Additionally, the majority of biomarkers has a higher abundance on PE, which may be due to the surface of non-degradable PE being colonized by microorganisms in soil as a common substrate. Therefore, we focused on the taxa with higher abundance on PLA and PBAT, than PE (Figures 4C,D), which were regarded as driving the variation in the different plastispheres. Four ASVs specifically found in PBAT were classified into the genera of *Ramlibacter* and *Cupriavidus*. Interestingly, the shared specific ASVs in the microbiota of PBAT and PLA all belonged to the genus *Ramlibacter* of the order *Burkholderiales*, which dominated the relative abundance at the order level for all four types of BDMs (Figure 2B).

### Discrepant Ecological Processes Dominated the Bacterial Assembly

**Figure 5:**
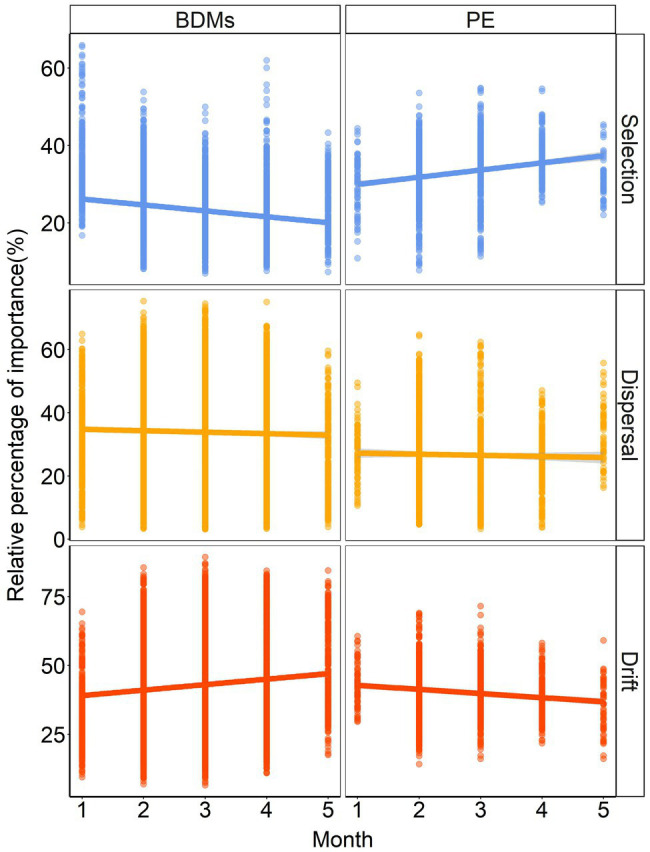
The assembly process of plastisphere microbiota based on infer Community Assembly Mechanisms by Phylogenetic-bin (iCAMP) in BDMs and PE.

To reveal the assembly mechanisms controlling the community diversity on the different mulches, we explored and quantified the relative importance of deterministic processes (selection) and stochastic processes (dispersal and others) in structuring the plastisphere microbial community with iCAMP (Figure 5). The results indicated that stochastic processes were the main contributors to the bacterial community assembly in both BDMs (70 ~ 80%) and PE (60 ~ 70%) during the 5 months sampling period. It was noteworthy that while the proportion of selection on BDMs and PE was similar (30%) in the initial month, they showed opposite trends over time in community assembly, especially in selection processes and drift processes. This suggested that although stochasticity dominated the community assembly of both BDMs and PE, they had completely different mechanisms. Additionally, in order to study the three ecological processes in temporal turnover between each sampling month, we further investigated the relative influence of the driving forces. It was clearly shown that the results demonstrated good consistency compared with five independent month samples. Stochastic processes dominated the temporal turnover of microbial community assembly in the plastisphere (Supplementary Figure S4). In general, despite the different trends over time, the above results revealed that the influence of stochastic processes ratio was much higher than that of selection processes in both BDMs and PE.

## Discussion

In order to determine the fate of both PE and BDMs in soils, it is necessary not only to characterize the species composition and assembly mechanism within the plastisphere, but also to reveal the succession of functional microbial taxa which drove the degradation. To date, studies on the plastisphere of PE and BDMs in soils have been relatively scarce (Ruthi et al., 2020; Zhou et al., 2021). In this study, we successionally surveyed bacteria in the plastisphere of PE, PLA, PBAT, and two mixed component mulches in the potted soils over a 5 months period. Exploring the temporal differences and similarities among conventional PE and four types of BDMs deepened our understanding of the functional potential and ecological impact of the plastisphere in soils.

Previous studies have shown that different plastic substrates could recruit various microbial communities to adhere and form biofilms. Meanwhile, the biofilms showed dynamic changes with the residence time of plastics in aquatic and soil environments (Jacquin et al., 2019; Wu et al., 2019; Ruthi et al., 2020). However, these studies usually demonstrated only the temporal changes of microbiota on single non-degradable plastics or BDMs individually, without determining whether the differences were caused by the residence time in soil or the plastic composition (Bandopadhyay et al., 2020; McGivney et al., 2020). Here, our study showed that composition of the plastic components and length of incubation were both important driving forces of microbiota dissimilarity and that both had significant effects (Table 1). When PE samples were considered, the difference in the composition of the plastics was greater than that of sampling month, while the difference in microbial community caused by incubation time of the four BDMs in soil was greater than that caused by different components (Table 1). Since these five types of plastics used in this study are widely applied in agricultural mulching, whether these results are applicable to other non-degradable plastics and BDMs remain to be investigated.

With the wide usage of BDMs in agriculture, the potential ecological risks of BDMs in soil have been of general interest (Bandopadhyay et al., 2020; Qin et al., 2021). Our results of species composition suggested that the microbiota of the five plastics were similar at the phylum level, but zooming in the order level, microbes of BDMs and PE showed significantly different patterns of composition and changes (Figure 2). Similar to our results, several studies have reported that the predominant bacterial orders of BDMs, *Burkholderiales* and *Pseudonocardiales*, were closely related to plastic degradation (Pérez-Pantoja et al., 2012; Ruthi et al., 2020). They have been observed to play a major role in the degradation of PLA. These suggested that BDMs were capable of enriching for specific plastic degrading bacteria, which was consistent with our results of the functional predictions (Figure 3). Some their genera have also been cultured to further explore potential metabolic pathways (Jeszeová et al., 2018). The protein RPA1511 isolated from *Rhodopseudomonas palustris* has the catalytic triad residues, which are important for the hydrolysis of both monoester and polyester substrates. It was considered to be critical for the hydrolysis of PLA and was active in catalyzing solid PLA with the production of lactic acid monomers, dimers, and larger oligomers (Hajighasemi et al., 2016; Gricajeva et al., 2021). Moreover, some functions related to the carbon, nitrogen, and sulfur metabolic pathways were revealed by FAPROTAX, which indicated that BDMs may affect the biogeochemical cycling of elements in agroecosystems. Considering the residual BDMs in the farmland as a carbon input, their ecological effects need to be assessed separately in terms of their degradation in the relatively short period and their continued accumulation in the long-term mulch. In particular, we were interested in the enriched functional taxa related to degradation and pathogenicity, as both of these groups are of wide concern and may have a direct impact on the ecological risk of agroecosystems. For example, *Xanthomonadales*, as an important and widely studied plant pathogen in agricultural production (Bayer-Santos et al., 2019), had been found on all plastic mulches. The above findings are supported by recent studies based on metagenomic and metatranscriptomic technologies in aquatic environments (Seeley et al., 2020; Bhagwat et al., 2021; Wright et al., 2021).

However, knowledge about the microbial taxa for several plastics remains limited, especially lack of qualitative comparison between non-degradable PE and BDMs (Bhagwat et al., 2021). Here, we particularly emphasized the comparative analysis using samples of PE as the control group, which provided us with an accurate assessment of whether the use of BDMs in agricultural production is effective in addressing the soil environmental contamination and ecological risks caused by PE. The response ratio results further suggested that distinct functional groups of degradation can be found on PE and BDMs though some taxa were shared, which could due to different degradation products and mechanisms (Figure 3). As found in previous studies, some cultured taxa were observed to have broad degradation capabilities of plastics, and some specific degrading genera may represent exclusive degradation mechanisms and metabolic pathways for one of plastic components (Pérez-Pantoja et al., 2012; Jeszeová et al., 2018; Butbunchu and Pathom-Aree, 2019). For example, various strains of *Pseudomonas* can secrete exopolysaccharides attachment to plastic mulch and have a broad range of enzymes, which are promising candidates for biodegradation of both PE and BDMs (Wilkes and Aristilde, 2017; Bandopadhyay et al., 2020), which can also be observed in the species composition of our study. In addition, it was surprising that BDMs seem to have an overall significantly higher (>95%) pathogenic functions for plant, animal, and human-associated pathogens compared to PE (Figure 3). This was consistent with the result of species composition at the order level (Figure 2B). Considering the degradation cycle of BDMs in soils, it is reasonable to assume that the residence of BDMs debris may cause more negative agricultural production impacts and serious ecological risks in the relatively short term (Moreno et al., 2017; Qin et al., 2021).

To further distinguish the representative taxa on BDMs in soils, a high-resolution RF model was carried out to identify individual bacterial species that could be sought to discriminate different plastic components. The results (Figure 4) indicated that most of the identified biomarkers were highly congruent for PLA/PE and PBAT/PE at the species (ASV) level. A possible explanation is that BDMs or their metabolites provide suitable ecological niches for the bacterial members that readily colonize the plastisphere. In addition, it was worth noting that all ASVs that specifically belonged to PLA and PBAT could be classified to the dominant order *Burkholderiales*. It has been widely reported that *Burkholderiales* had a specialized ability for decomposing recalcitrant polyaromatic hydrocarbons or act as a plant or animal pathogen (Pérez-Pantoja et al., 2012; Sasaki et al., 2021). This was consistent with the results of species classification and functional prediction (Figures 1, 2). For the temporal heatmap, it was obvious that the biomarkers of PE and BDMs varied greatly with sampling month. This may be due to the fact that the effects brought by the plastic components were reflected not only in the differentiation of the entire bacterial community (Figure 1), but also in the impact on these specific biomarker species.

Disentangling the assembly mechanisms of biofilm microbial communities is a longstanding issue of ecologists. To date, studies have explored the dynamics of microbial communities inherited from conventional non-degradable plastics in aqueous environments by means of null modeling-based or neutral theory-based approaches. Most studies demonstrated the dominant role of stochastic processes in plastisphere microbiota (Sun et al., 2021; Zhang et al., 2021; Zhu et al., 2021), and a few suggested a higher influence of deterministic ecological processes (Hou et al., 2021; Li et al., 2021a). None of previous studies presented the microbial assembly of BDMs in soils. Although our results on species composition and function tended to suggest strong selection as the driver of community construction, the iCAMP results demonstrated a higher proportion of stochastic processes (dispersal plus drift) than deterministic processes (selection) for both BDMs and PE (Figure 5; Supplementary Figure S4). A smaller selective role may imply that the plastisphere microbial community was more resistant to changes in plastic components and environmental factors. Many previous studies have found that properties of plastics and environmental factors largely affected the composition and assembly mechanism of microbial communities (Li et al., 2021a; Sun et al., 2021). One of the possible explanations for the lower selection observed in our study is that the samples were only collected in our potted soils. The scale of sampling may influence the importance of stochastic and deterministic processes in shaping microbial communities (Chase, 2014; Zhou and Ning, 2017). It was reported that narrow scales tended to maintain a lower level of environmental heterogeneity, which may reduce the impact of selection and increase the role of stochastic processes (Garzon-Lopez et al., 2014; Wang et al., 2020b; Sun et al., 2021). These studies corroborated with our findings on BDMs. Therefore, sampling scale effects need to be considered when interpreting the assembly patterns of microbial communities on plastic in future studies.

Another interesting result was the opposite trends of bacteria on BDMs and PE mulches in the selection and drift processes over time (Figure 5; Supplementary Figure S4). The potential reason of their opposite trends may be the temporal scale effects of low migration and preferential colonized selection effects within plastisphere communities in soil. The proportion of selection on BDMs and PE was similar in the initial month, which may be due to the fact that BDMs had not yet started to degrade and both of them were present as common adherents. Thereafter, the selection stress on PE mulch consistently increased over time (Figure 5). This was possibly attributed to the microbial preferential colonization and selection effects that emerged in PE as a non-degradable stable plastic, resulting in enhanced selection (Wang et al., 2020a; Li et al., 2021a; Sun et al., 2021). In contrast, with the continuous degradation and fragmentation of BDMs over time, the trend of the microbial survival space (colonized area provided by the substrate) and proportion of selection process were consistently decreasing (Sander, 2019; Sintim et al., 2020). It is probably due to the spatial separation of the plastisphere microecology and niche caused by degradation, causing an increase in the stochastic process (Zhou and Ning, 2017; Sun et al., 2021). This may further elucidate the deep mechanism by which non-degradable PE and BDMs have different patterns of community construction. Moreover, our findings also indicated that understanding the pattern of temporal turnover was of great significance in revealing the community assembly of plastisphere.

## Conclusion

In this work, we focused on five plastic mulches (especially BDMs) in soil and performed a time-series pot soil trial to track the changes in species composition and function of plastic-associated microbiota during degradation. Samples with similar plastic composition showed more similar changes at the phylum and order levels, while BDMs and PE were capable of enriching for specific plastic degrading bacteria which may imply that they had different degradation mechanisms. For future studies, combining multi-omics approaches, such as macroproteomics, metaproteomics, and culturomics, together may be a trend to provide comprehensive information on the biodegradation mechanisms and metabolic pathways. Enriched degradation-related and potential pathogen-related functional groups were discussed in this report, which provided a reference for assessing the potential ecological risk of BDMs debris in agricultural fields. BDMs and PEs showed a predominance of stochastic processes and displayed opposite patterns of community assembly over time. Sampling and temporal scale effects demonstrated the possible underlying mechanisms of the phenomenon. Therefore, future studies need to take these factors into account when focusing on the succession and assembly mechanisms in the plastisphere of BDMs. More soil types and locations also need further investigation in the experimental design to exclude the influence of discrepancies in physicochemical properties on the results.

## Data Availability Statement

The datasets presented in this study can be found in online repositories. The names of the repository/repositories and accession number(s) can be found in the article/Supplementary Material.

## Author Contributions

ZJ, DJ, and YD conceived and designed the experiments. ZJ performed the experiments. ZJ, XD, KF, SL, and SG analyzed the data. ZJ and YD wrote the paper. All authors read and approved the final manuscript.

## Funding

This study was supported by the National Natural Science Foundation of China (nos. 31861133002 and 41977122).

## Conflict of Interest

The authors declare that the research was conducted in the absence of any commercial or financial relationships that could be construed as a potential conflict of interest.

## Publisher’s Note

All claims expressed in this article are solely those of the authors and do not necessarily represent those of their affiliated organizations, or those of the publisher, the editors and the reviewers. Any product that may be evaluated in this article, or claim that may be made by its manufacturer, is not guaranteed or endorsed by the publisher.

## Footnotes

^1^ http://mem.rcees.ac.cn:8080

^2^ http://ieg3.rccc.ou.edu:8080
