# Supplementary material for: The Succession of Bacterial Community Attached on Biodegradable Plastic Mulches During the Degradation in Soil
Source: Front Microbiol. 2021 Dec 24;12:785737. doi: 10.3389/fmicb.2021.785737 (PMC8762578; doi:10.3389/fmicb.2021.785737)
Supplement: Supplementary file 1 [file Data_Sheet_1.docx]

Supplementary Material

# Supplementary Figures

**
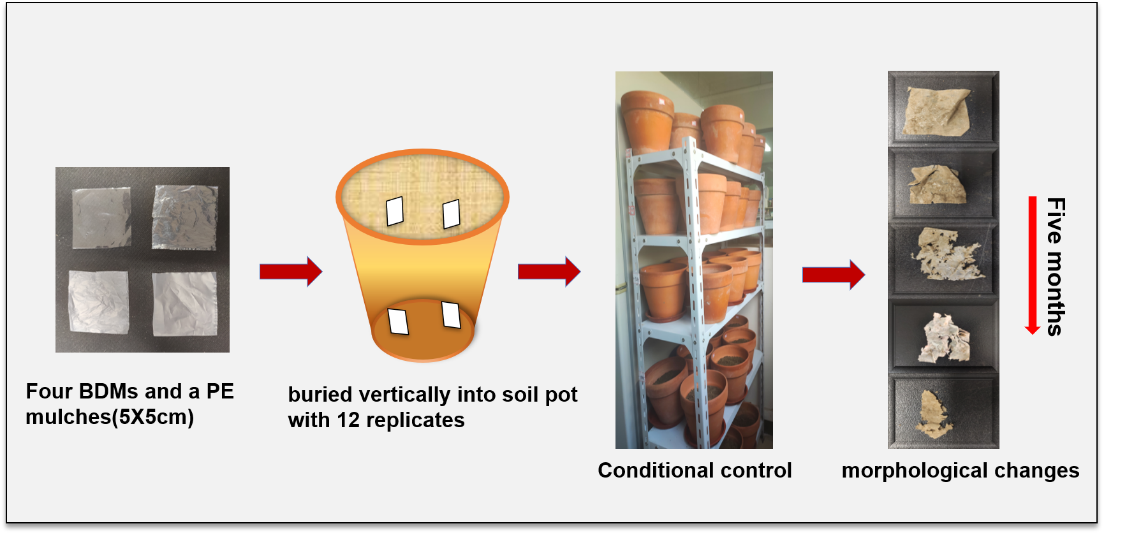
**

**Supplementary Figure 1.** Flowchart of experimental design. Firstly, five types of mulches were cut into square pieces of 5 cm^2^ and steriled. Then they were buried vertically into separate pots and all treatments were performed in 12 replicates. The pots were put in the condition-controlled room at 23 °C ± 3°C with 15 ± 5% air moisture during the incubation period. Finally, the mulch samples were carefully collected from every pot for five consecutive months. The BDMs had undergone obvious morphological changes during this incubation period.


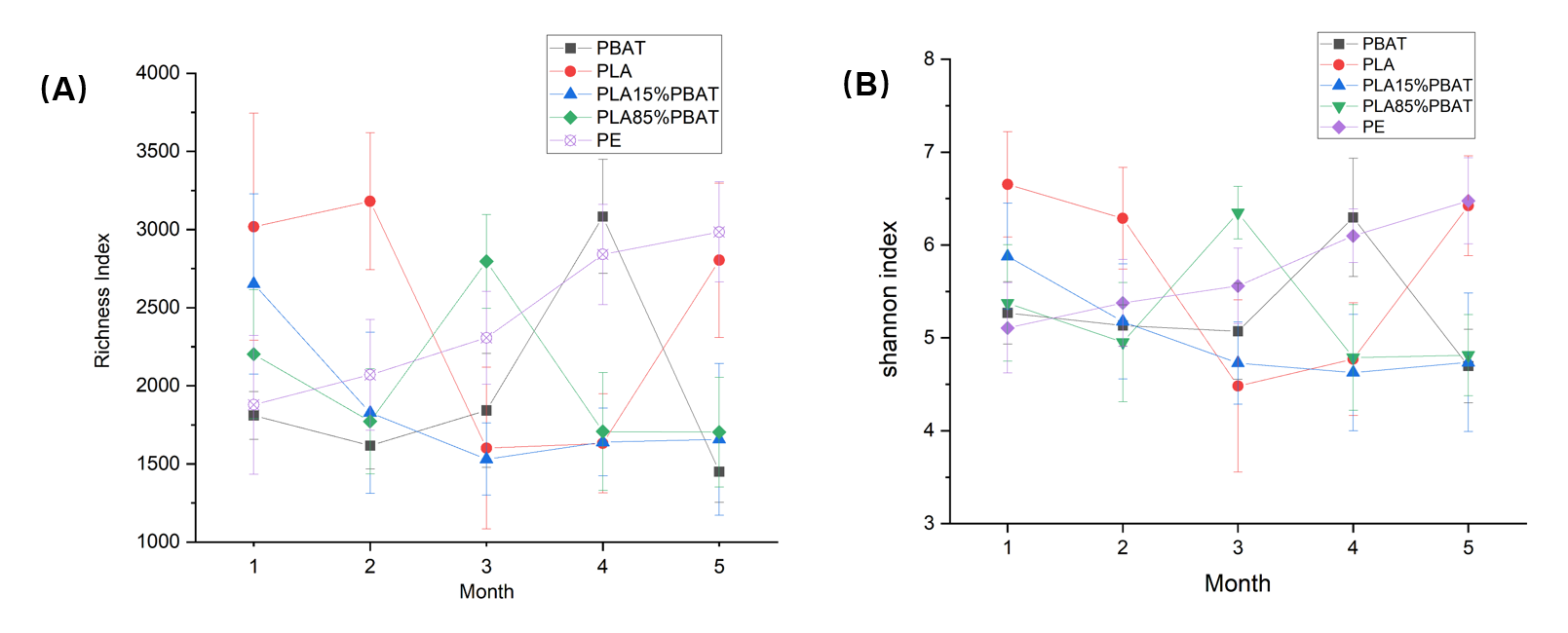


**Supplementary Figure S2.** α- diversities for five kinds of plastic mulches over the course of five sampling months. Variations in microbial Shannon index **(A)** and richness index **(B)** among five kinds of plastic mulches during five months.


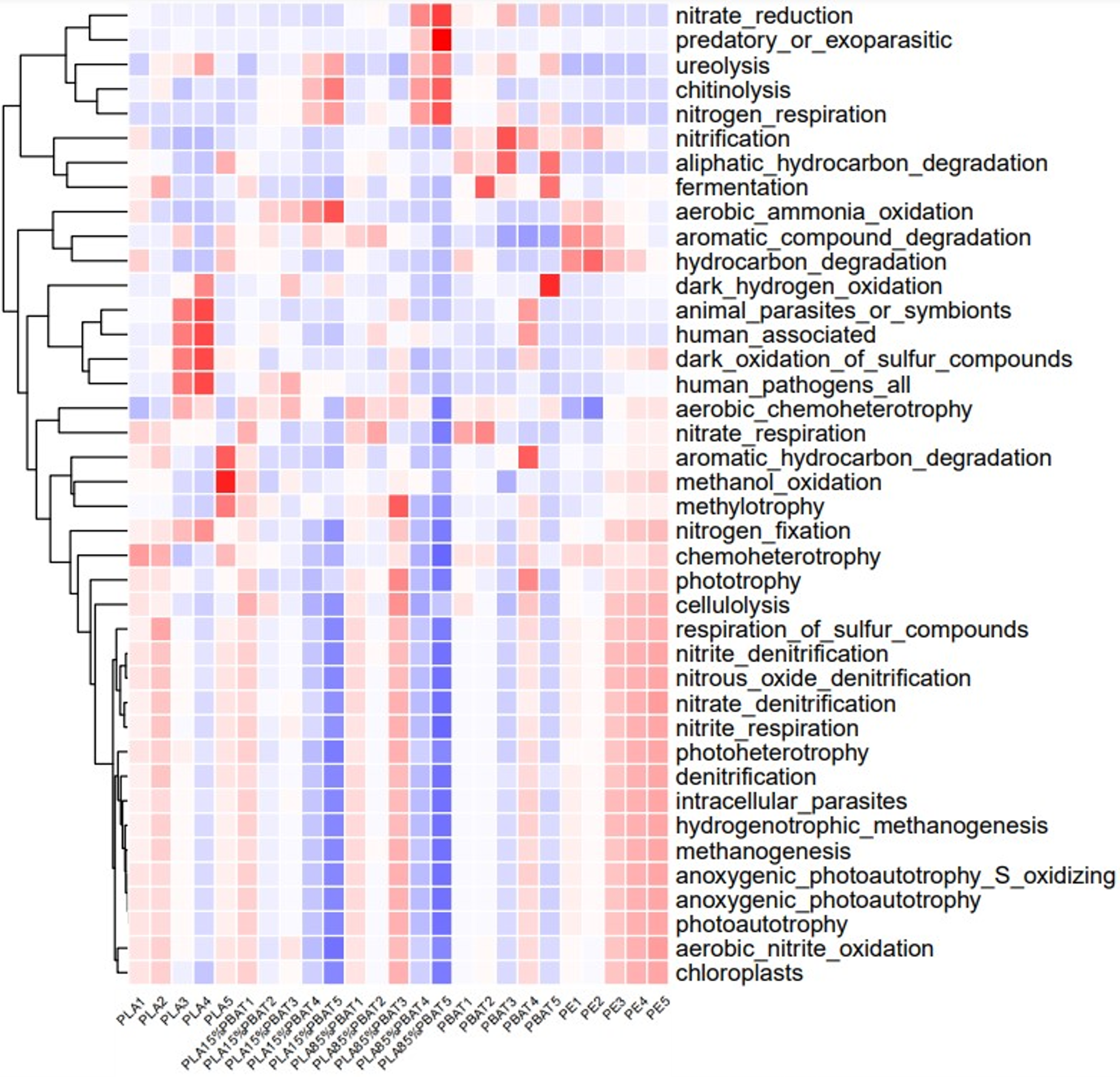


**Supplementary** **Figure S3.** The top 40 functional annotations of prokaryotic taxa by Functional Annotation of Prokaryotic Taxa (FAPROTAX) for the five plastics.


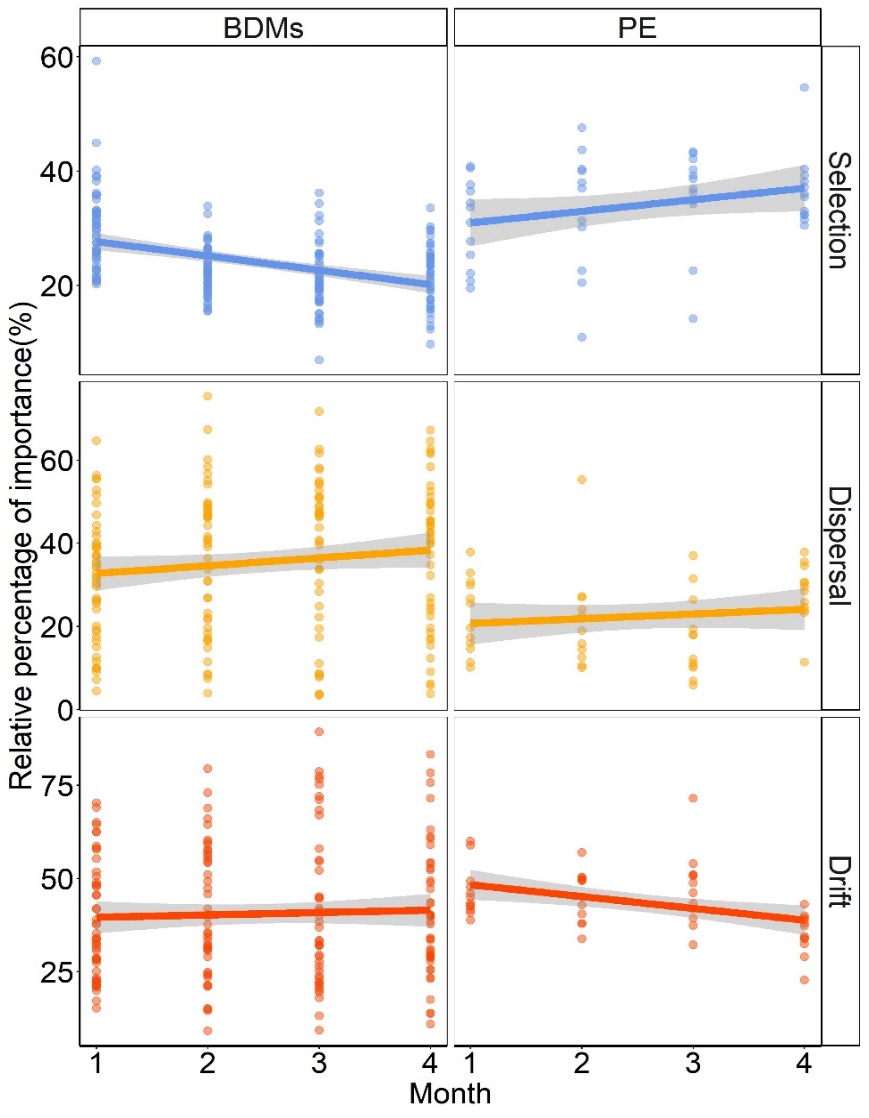


**Supplementary Figure S4.** The three ecological processes in temporal turnover between each adjacent sampling month during five months based on infer Community Assembly Mechanisms by Phylogenetic-bin (iCAMP) in BDMs and PE. For example, the value in 1 represents the ratio of the corresponding samples for the first and second sampling months.

**the Code availability:** Scripts employed in the computational analyses of the random forest and community assembly are available at

https://github.com/yedeng-lab/the_microbiota_of_biodegradable_plastic.
